# Interaction of Lipids, Mean Platelet Volume, and the Severity of Coronary Artery Disease Among Chinese Adults: A Mediation Analysis

**DOI:** 10.3389/fcvm.2022.753171

**Published:** 2022-01-31

**Authors:** Yao Yao, Xiaoye Li, Zi Wang, Qiuyi Ji, Qing Xu, Yan Yan, Qianzhou Lv

**Affiliations:** ^1^Department of Pharmacy, Zhongshan Hospital, Fudan University, Shanghai, China; ^2^Department of Cardiology, Zhongshan Hospital, Fudan University, Shanghai, China

**Keywords:** mediation analysis, mean platelet volume, serum lipid, coronary artery disease, Gensini score

## Abstract

**Objective:**

Currently, coronary artery disease (CAD) is regarded as one of the leading global disease burdens. Evidence proved that platelet activation in dyslipidemia induced CAD, however, their interaction has not been well-established *in vivo*. This study aims to assess the mediation effects of mean platelet volume (MPV) in lipids and the severity of CAD.

**Methods:**

We prospectively enrolled 5,188 consecutive subjects who underwent coronary angiography between 2015 and 2020. Participants were grouped according to their CAD events, which was defined as stenosis ≥50% in at least one coronary artery, and whose severity was evaluated by the Gensini score (GS). A lipid index was drawn by principal component analysis to weight related lipid parameters including total cholesterol (TC), low density lipoprotein cholesterol (LDL-C), high density lipoprotein cholesterol (HDL-C), non-HDL-C, apolipoprotein (apo) A1 B. The interaction of lipids and MPV in atherosclerosis was evaluated by the mediation analysis.

**Results:**

Lipid index increased with elevated GS irrespective of statin status (not on statin: β = 0.100, *p* < 0.001; on statin: β = 0.082, *p* < 0.001). Multiple linear regression indicated positive correlation between MPV and GS after adjustment (β = 0.171, *p* < 0.001). Subjects in the highest MPV tertile had higher levels of atherogenic lipid parameters and lipid index (*p* < 0.001). The adjusted odds ratios were greater among individuals undergoing statin medications who had high GS and higher MPV levels by elevated lipid index tertiles [1.168 (0.893–1.528) vs. 2.068 (1.552–2.756) vs. 1.764 (1.219–2.551)]. The combination of lipid index and MPV provided better prediction for high GS than individual lipid index or MPV, as shown by receiver-operating characteristic (ROC) curves (areas under ROC curves were 0.700 and 0.673 in subjects on or not on statin treatment, respectively). Significantly, mediation analysis revealed the mediation interaction of lipid index on GS by MPV, whose effect size reached 20.71 and 20.07% in participants with or without statin medications.

**Conclusion:**

The increased risk of dyslipidemia on CAD was partly enhanced by elevated MPV levels, whose mediating effect was around 20%.

## Introduction

Coronary artery disease (CAD) is the leading cause of morbidity and mortality worldwide. With the rapid development of medical science, the mortality attributable to CAD has been declining for more than 4 decades (1); however, the incidence of CAD continues to rise with an estimated incidence of 11 million cases in China in 2017 (2) and 605 thousand new heart attacks annually in the United States (3), in part because risk factors remain alarmingly high.

The disease mainly ensues from atherosclerotic plaques or occlusions of the coronary arteries (4). Hyperlipidemia is a major risk factor for the development of CAD, with which individuals typically have elevated plasma low-density lipoprotein cholesterol (LDL-C) concentration as well as decreased circulating high-density lipoprotein cholesterol (HDL-C) level (5). High concentration of LDL possesses the ability to impair endothelium and induce oxidation, leading to atheromatous plaque formation and the narrowing of lumen (6). Other proatherogenic lipoproteins include triglycerides (TG), total cholesterol (TC), apolipoprotein (apo) B, etc. In contrast, HDL is a strongly inverse predictor of CAD with its properties to mediate cholesterol efflux from macrophages (7). ApoA1 is the primary protein component of HDL particles and also plays an antiatherogenic role (8).

The occurrence of CAD is dependent on conventional risk factors; however, more than 50% of the cases can be ascribed to non-traditional risk factors, and its underlying mechanisms have not been fully understood (9). Inflammatory responses attributed to lipid accumulation in the intima of coronary arteries cause atherosclerosis plaque instability, stimulating the activation and aggregation of platelets (10, 11). Platelet activation leads to morphological alterations and the formation of pseudopodia with the release of granular contents (12); meanwhile it plays a pathogenic role in triggering acute coronary syndromes, which leads to mortality in many patients with CAD (13). However, platelet function tests are cost-intensive and time-consuming, thereby limiting its use in clinical practice (14). Mean platelet volume (MPV) is a measurement of platelet size and it is of low-cost and readily available from fasting blood samples, as recommended by ICSH (15). Various studies found an association between MPV and CAD or myocardial infarction (16–18). Hence, MPV may represent a risk factor in CAD.

Previous studies implied that atherogenic lipids enhanced platelet responsiveness, whereas HDL opposed their activating capacity on platelets *in vitro* (19, 20). Notwithstanding, medical data in respect to the interaction between lipids and MPV in CAD is largely inaccessible. In accordance with the preceding discussion, we hypothesized that the interaction between lipids and MPV might produce synergistic effects in coronary atherosclerosis.

Consequently, in this study with a Chinese cohort of 5,188 individuals, we aimed to explore the potential role of MPV in clinical practice by analyzing the interaction of lipids and MPV in the severity of coronary artery atherosclerosis and assessing the mediation effects of MPV.

## Methods

### Study Design and Population

The study complied with the principles expressed in the Declaration of Helsinki and was approved by Medical Ethics Committee of Zhongshan Hospital. All participants gave written informed consent for the data included in this study.

We enrolled 5,188 consecutive subjects who underwent coronary angiography due to suspected CAD in our institution from January 2015 to December 2020. Exclusion criteria were patients with age <18 years; with no lipid parameters, MPV, or other design data of this study available; antiplatelet medication history within 3 months prior to the admission; medication history of lipid-lowering drugs for subjects without in-hospital statin prescription; a history of previous revascularization (percutaneous intervention or coronary artery bypass surgery); fatal heart failure; cardiac valve disease; arrhythmia; infectious or systematic inflammatory disease; significant hematologic disorders; thyroid dysfunction; severe liver or renal dysfunction; or malignancies.

Complete demographic, laboratory, and angiographic data were obtained from all subjects and included in a dedicated database. CAD was defined as stenosis ≥50% in one or more coronary arteries, assessed by at least two experienced interventional cardiologists. Individuals whose coronary angiogram did not achieve the diagnostic criteria of CAD were included in the non-CAD group. Besides, we defined 1-vessel, 2-vessel, and 3-vessel disease according to the number of major coronary vessels with stenosis ≥50%. Hypertension was defined when systolic and/or diastolic blood pressure was ≥140 and/or ≥90 mmHg, respectively, on multiple different occasions or when patients self-reported the prior diagnosis or currently took anti-hypertensive drugs. Diabetes mellitus was defined as fasting glucose ≥ 7.0 mmol/L repeatedly, hemoglobin A1c (HbA1c) ≥ 6.5%, or current use of antidiabetic medications. Current smokers were ascertained when individuals currently smoked at least once a day for over 1 year or quitted smoking less than half a year.

### Laboratory and Clinical Analyses

Venous blood samples were collected after a 12-h overnight fast on the second day of admission for lipids, blood routine, glucose, liver, and renal function from all subjects. Lipid parameters including TC, TG, LDL-C, HDL-C, apoA1, and apoB were measured using a Hitachi 7600-120 automated biochemistry analyzer (Hitachi, Tokyo, Japan). Non-HDL-C values were calculated by subtracting HDL-C from TC. A Sysmex XS 500i hematology analyzer (Sysmex, Kobe, Japan) was used to determine complete blood counts, including MPV. All other included biomarkers were analyzed consistent with the standard operating protocol and the manufacturers' instructions.

Severity of coronary atherosclerosis was quantified by the Gensini score (GS) (21). In detail, points were first assigned according to the degree of coronary artery stenosis, which was 1 point for stenosis of 1–25%, 2 points for 26–50%, 4 points for 51–75%, 8 points for 76–90%, 16 points for 91–99%, and 32 points for complete occlusion. Second, the previous points were multiplied by a factor represented by the segment of corresponding coronary arteries, of which the specific points were 5 points for the left main coronary, 2.5 points for the proximal segment of left anterior descending coronary artery (LAD) and the proximal segment of the circumflex artery (LCx); 1.5 points for mid-segment of the LAD; 1 point for the right coronary artery, the distal segment of the LAD, the posterolateral artery, and the obtuse marginal artery; and 0.5 point for all others. Finally, GS was the sum of the points above.

### Statistical Analysis

Kolmogorov–Smirnova test was applied to all parameters, and then homogeneity of variance was assessed by Levene's test. Descriptive statistics for continuous data were expressed as means and standard deviations (mean ± SD) or median with interquartile range (IQR) and were compared between groups using two-tailed Student's *t*-test or Mann–Whitney test depending on the distribution of data. For multiple group comparisons, one-way ANOVA or Kruskal–Wallis with *post-hoc* Bonferroni or Games–Howell multiple comparisons were utilized. Categorical variables were presented as frequencies and percentages using a chi-square test or Fisher's exact probability test.

We performed a principal component analysis in TC, LDL-C, HDL-C, non-HDL-C, apoA1, and apoB and saved the factor scores (i.e., standardized, weighted sum score) for each principal component. The lipid index was generated by summing up each factor score. Simple linear regression analyses were performed to assess the associations between lipid index and GS.

Lipid profiles, platelet parameters and other potential risk factors of CAD were entered into univariate linear regression analyses. Variables with *p* < 0.05 or known risk factors in univariate analyses were then included for multivariate regression analyses to determine the independent relationship between MPV and GS. Due to skewed distribution, GS was logarithmically transformed. We checked multicollinearity among the variables using the variance inflation factor (VIF). The VIF for each variable was <10 and it was accepted in the multiple linear regression.

The interaction of lipid index tertiles and MPV tertiles in high GS subjects was tested by multivariate logistic models with odds ratios (OR) and 95% confidence intervals (95% CI), adjusted for age and gender. Furthermore, we performed receiver-operating characteristic curves (ROC) for lipid index, MPV, and lipid index+MPV to identify the prediction of high GS by comparing areas under each curve (AUC).

We conducted mediation analyses with the computational tool PROCESS version 3.5.1 in SPSS to determine MPV mediation effect with adjustment for age and gender. In this model, the independent variables were atherogenic lipids including TC, LDL-C, non-HDL-C, apoB, and lipid index. MPV was regarded as the mediator. The dependent variable was GS. The effect size of the mediator was calculated by the ratio of indirect effect to total effect. To test the potential mediation effect, we reported bootstrap support values with 95% CI obtained from 1,000 iterations (22).

All statistical analyses were performed using SPSS 26.0 (WPSS Ltd., Surrey, UK). Probability values of <0.05 were statistically significant.

## Results

### Baseline Clinical Characteristics

**Figure 1:**
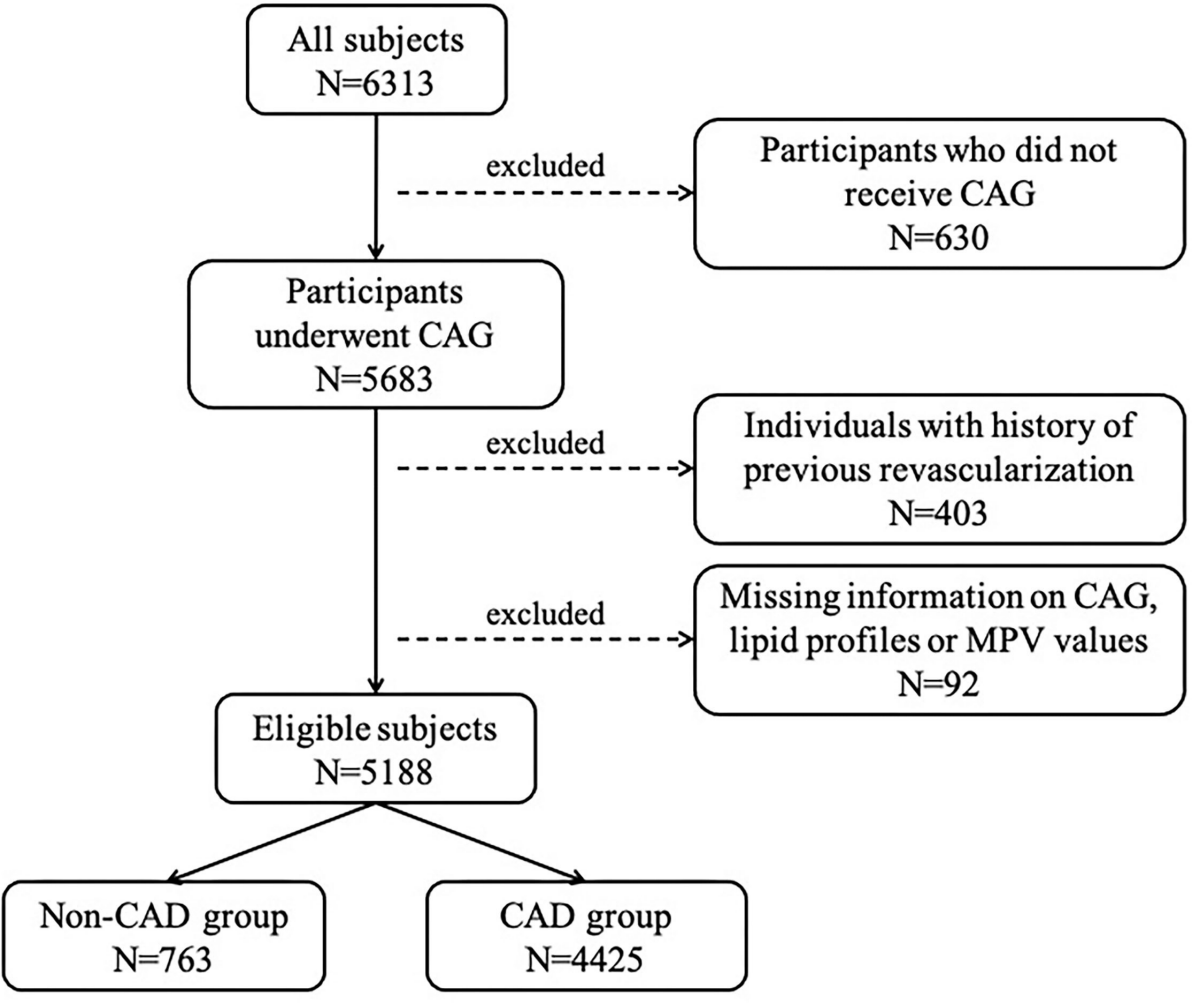
Flow chart depicting participants enrollment procedure. CAG, coronary angiography; MPV, mean platelet volume; CAD, coronary artery disease.

From January 2015 to December 2020, a total of 6,313 individuals with suspected CAD intending to receive coronary angiography were consecutively recruited in the department of Cardiology, Zhongshan Hospital, with 5,188 participants ultimately enrolled and assigned to the CAD group (*n* = 4,425, 85.3%) or non-CAD group (*n* = 763, 14.7%) based on diagnosis (Figure 1). Baseline characteristics of total subjects are shown in Table 1. The average age of this population was 64.61 ± 10.26 years and consisted of 74.1% men. The median GS level was 32 (14–60) in the CAD group. Compared to non-CAD group, patients with CAD had significantly higher levels of MPV and lower levels of overall lipid variables, with the exception of TG due to greater rate of statin use.

**Table 1:** Baseline demographic and clinical characteristics of study subjects who underwent CAG.

**Characteristics**	**Overall** **(*n* = 5,188)**	**CAD group** **(*n* = 4,425)**	**non-CAD group** **(*n* = 763)**	***P*-value**
**Risk factors**				
Age (years)	64.61 ± 10.26	64.94 ± 10.40	62.65 ± 11.57	<0.001
Male, *n* (%)	3,842 (74.1%)	3,355 (75.8%)	487 (63.8%)	<0.001
Smoking, *n* (%)	2,640 (50.9%)	2,419 (54.7%)	221 (29.0%)	<0.001
BMI (kg/m^2^)	24.71 ± 3.58	24.72 ± 3.58	24.68 ± 3.60	0.913
Hypertension, n (%)	2,297 (44.3%)	2,026 (45.8%)	271 (35.5%)	<0.001
Diabetes mellitus, n (%)	1,743 (33.6%)	1,565 (35.4%)	178 (23.3%)	<0.001
**Laboratory values**				
HbA1c, mmol/mol	6.40 ± 1.26	6.44 ± 1.26	6.15 ± 1.2	<0.001
hsCRP, mg/L	1.58 [0.61–5.53]	1.57 [0.60–5.27]	1.64 [0.67–6.72]	0.014
FIB (g/L)	288.80 [246.90–347.50]	289.15 [247.20–347.30]	285.80 [243.40–353.90]	0.595
WBC, 10^9^/L	6.81 [5.56–8.44]	6.80 [5.55–8.35]	6.86 [5.65–9.26]	<0.001
PLT, 10^9^/L	207.50 ± 59.87	207.09 ± 59.98	209.90 ± 59.23	0.231
PCT, %	0.23 ± 0.057	0.22 ± 0.057	0.23 ± 0.056	0.083
MPV, fL	10.93 ± 1.06	10.98 ± 1.08	10.61 ± 0.94	<0.001
PDW, %	12.90 ± 2.38	12.91 ± 2.40	12.87 ± 2.25	0.654
cTnT, ng/mL	0.017 [0.008–0.26]	0.017 [0.008–0.20]	0.027 [0.007–0.77]	<0.001
TC, mmol/L	3.90 ± 1.07	3.85 ± 1.06	4.16 ± 1.07	<0.001
TG, mmol/L	1.88 ± 1.43	1.90 ± 1.40	1.79 ± 1.57	0.053
LDL-C, mmol/L	2.39 ± 0.94	2.35 ± 0.94	2.62 ± 0.93	<0.001
HDL-C, mmol/L	1.09 ± 0.315	1.08 ± 0.35	1.17 ± 0.34	<0.001
non-HDL-C, mmol/L	2.80 ± 1.05	2.77 ± 1.05	2.99 ± 1.04	<0.001
apoA1, g/L	1.26 ± 0.26	1.26 ± 0.25	1.30 ± 0.28	<0.001
apoB, g/L	0.77 ± 0.245	0.76 ± 0.25	0.81 ± 0.24	<0.001
**Incidence of coronary atherosclerosis**				
1-vessel disease, *n* (%)		1,866 (42.2%)		
2-vessel disease, *n* (%)		1,473 (33.3%)		
3-vessel disease, *n* (%)		1,086 (24.5%)		
GS		32 (14–60)		
**Cardiovascular medication use**			
Beta-blocker, *n* (%)	2,533 (48.8%)	2,282 (51.6%)	251 (32.9%)	<0.001
ACEI/ARB, *n* (%)	1,101 (21.2%)	1,014 (22.9%)	87 (7.9%)	<0.001
Statin, *n* (%)	3,660 (70.5%)	3,228 (72.9%)	432 (56.6%)	<0.001

*Data were expressed as n (%), median [quartiles) or mean ± standard deviation. P < 0.05 were statistically significant. CAG, coronary angiography; CAD, coronary artery disease; BMI, body mass index; hsCRP, high sensitivity C-reactive protein; FIB, fibrinogen; WBC, white blood cell count; PLT, platelet count; PCT, plateletcrit; MPV, mean platelet volume; PDW, platelet distribution width; cTnT, cardiac troponin T; TC, total cholesterol; TG, triglyceride; LDL-C, low density lipoprotein-cholesterol; HDL-C, high-density lipoprotein-cholesterol; non-HDL-C, non-high-density lipoprotein-cholesterol; apo, apolipoprotein; GS, Gensini score; ACEI, angiotensin converting enzyme inhibitors; ARB, angiotensin receptor blocker*.

### Association of Lipids With GS

**Figure 2:**
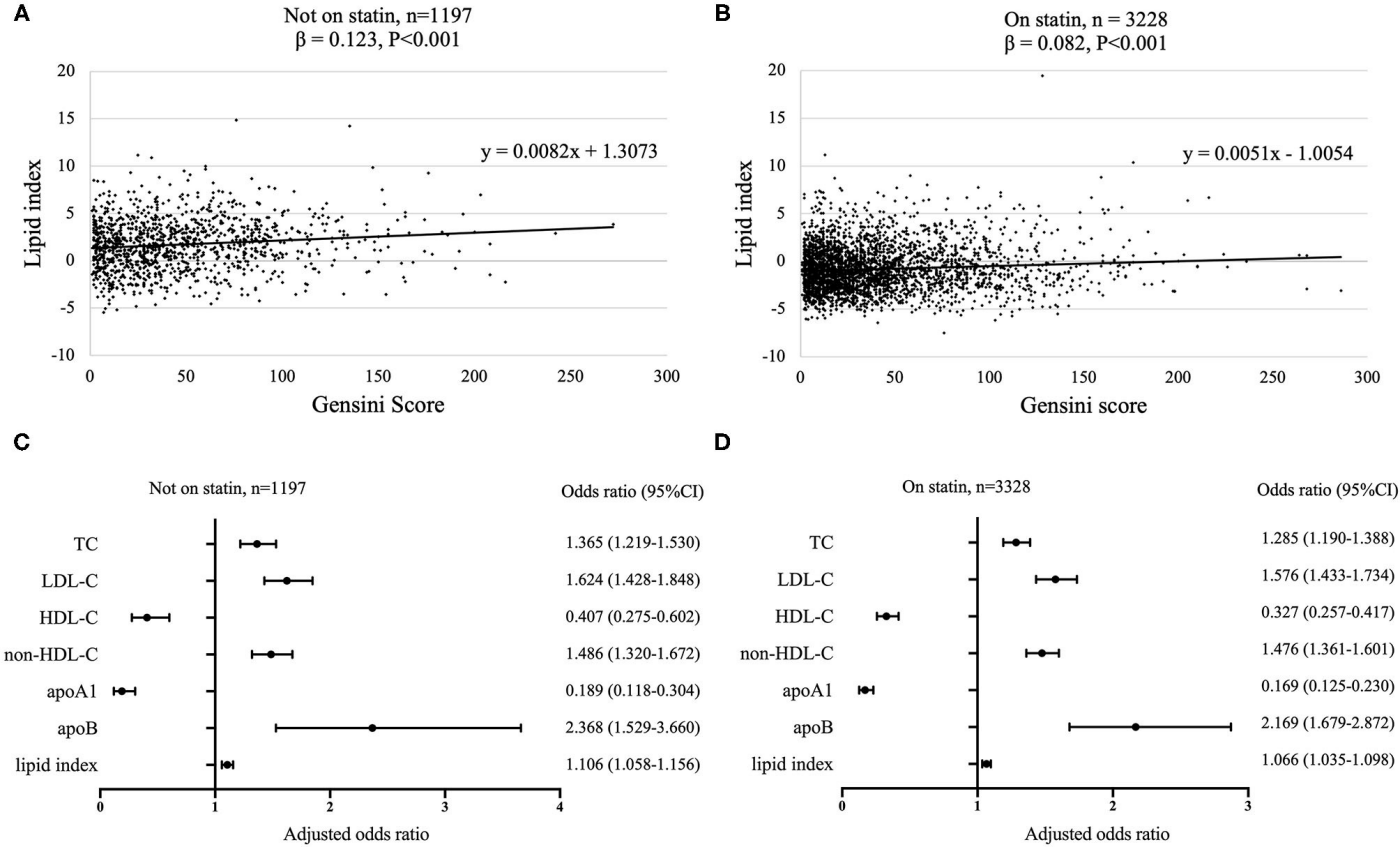
Association between lipids and GS according to statin status. Simple linear regression analyses were used to show the association between lipid index and GS [**(A)** not on statin; **(B)** on statin]. Relationship between lipids and high GS was assessed by multivariate logistic regression models with adjustment for age and gender [**(C)** not on statin; **(D)** on statin]. 95% CI, 95% confidence interval; GS, Gensini score; TC, total cholesterol; LDL-C, low density lipoprotein-cholesterol; HDL-C, high-density lipoprotein-cholesterol; non-HDL-C, non-high-density lipoprotein-cholesterol; apo, apolipoprotein.

To better analyze the data, the GS of patients with CAD was divided by tertiles [low (≤19, *n* = 1,495), median (20–48, *n* = 1,490), and high (≥49, *n* = 1,440)], of which the profiles of demographics and baseline assessments are provided in the Supplementary Material. Statin medications may have an impact on the lipid level; thus we subgrouped the cohort according to statin status. Proatherogenic lipid profiles of TC, LDL-C, non-HDL-C, and apoB increased with elevated GS tertiles regardless of statin use status (*p* < 0.001). Also, we revealed the negative associations between HDL-C and apoA1 with GS (*p* < 0.001) (Table 2). A lipid index was extracted from TC, LDL-C, HDL-C, non-HDL-C, apoA1, and apoB by principal component analysis. In this model, the first and second principal components captured 90.87% of the variation contained within the 6 variables, in which the first component accounted for 61.86% of variance, whereas the second component explained 29.01% variance (Table 3). The formulation of lipid index in this model was as follows: 4.949^*^factor score 1 +2.321^*^factor score 2. Positive correlations of lipid index and GS were indicated in simple linear regression analysis (not on statin: coefficient, 0.123, *p* < 0.001; on statin: coefficient, 0.082, *p* < 0.001, Figures 2A,B). Figures 2C,D showed the relationship between lipid and high GS by multivariable logistic regression.

**Table 2:** Statin-stratified lipid levels by GS with adjustment for age and gender.

	**GS tertiles**			***P*-value**
	**Low (≤19)** **(*n* = 1,495)**	**Median (20–48) (*n* = 1,490)**	**High (≥49)** **(*n* = 1,440)**	
Not on statin (*n* = 1,197)				
TC, mmol/L	4.36 ± 0.97	4.48 ± 1.06	4.78 ± 1.14	<0.001
TG, mmol/L	2.00 ± 1.09	1.89 ± 1.20	1.97 ± 1.28	0.460
LDL-C, mmol/L	2.81 ± 0.85	2.99 ± 0.97	3.32 ± 1.01	<0.001
HDL-C, mmol/L	1.13 ± 0.31	1.13 ± 0.23	1.05 ± 0.26	<0.001
non-HDL-C, mmol/L	3.24 ± 0.92	3.35 ± 1.06	3.73 ± 1.13	<0.001
apoA1, g/L	1.34 ± 0.26	1.29 ± 0.26	1.22 ± 0.24	<0.001
apoB, g/L	0.86 ± 0.22	0.91 ± 0.25	0.99 ± 0.27	<0.001
On statin (*n* = 3,228)				
TC, mmol/L	3.49 ± 0.85	3.55 ± 0.87	3.74 ± 1.05	<0.001
TG, mmol/L	1.82 ± 1.40	1.89 ± 1.38	1.95 ± 1.64	0.111
LDL-C, mmol/L	1.95 ± 0.69	2.07 ± 0.74	2.27 ± 0.87	<0.001
HDL-C, mmol/L	1.14 ± 0.34	1.06 ± 0.28	1.01 ± 0.29	<0.001
non-HDL-C, mmol/L	2.36 ± 0.81	2.49 ± 0.87	2.73 ± 1.03	<0.001
apoA1, g/L	1.31 ± 0.26	1.24 ± 0.23	1.18 ± 0.25	<0.001
apoB, g/L	0.66 ± 0.19	0.70 ± 0.20	0.75 ± 0.23	<0.001

*A general linear model was performed after the adjustment for age and gender according to the use of statin status. Data were expressed as mean ± standard deviation. GS, Gensini score; TC, total cholesterol; LDL-C, low density lipoprotein-cholesterol; HDL-C, high-density lipoprotein-cholesterol; non-HDL-C, non-high-density lipoprotein-cholesterol; apo, apolipoprotein*.

**Table 3:** The factor matrix for the component analysis.

**Principal item**	**Component 1**	**Component 2**
TC	0.887	0.431
LDL-C	0.896	0.289
HDL-C	−0.213	0.927
non-HDL-C	0.966	0.161
apoA1	−0.103	0.935
apoB	0.954	0.137

*Component 1 showed a strong positive correlation with TC, LDL-C, non-HDL-C and apoB. Component 2 was strongly and positively related with HDL-C and apoA1. TC, total cholesterol; LDL-C, low density lipoprotein-cholesterol; HDL-C, high-density lipoprotein-cholesterol; non-HDL-C, non-high-density lipoprotein-cholesterol; apo, apolipoprotein*.

### Association of MPV With GS

**Figure 3:**
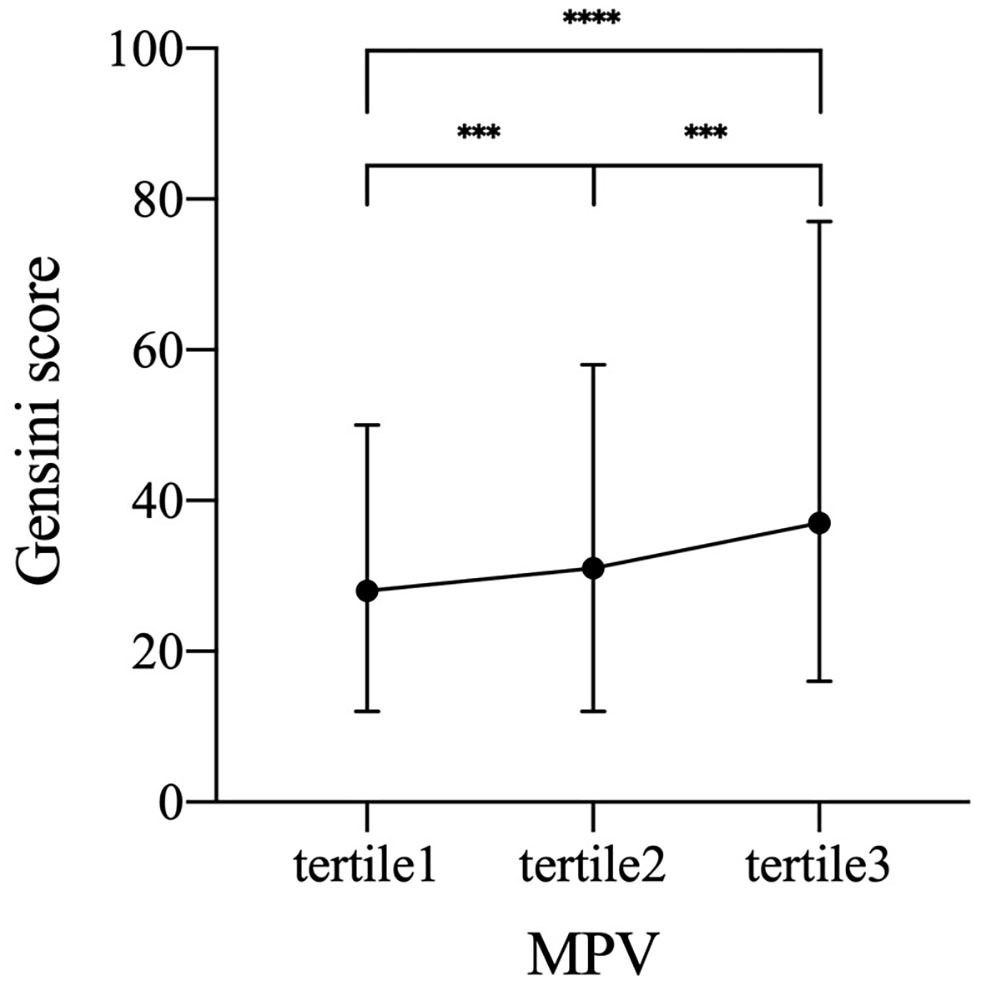
Increased GS by MPV tertiles. Symbols and error bars represent the medians and interquartile ranges, respectively due to skewed distribution of GS. ***p < 0.001 and ****p < 0.0001. GS, Gensini score; MPV, mean platelet volume (tertile 1, MPV ≤ 10.4 fL; tertile 2, MPV 10.5–11.4 fL; tertile 3, MPV≥11.5 fL).

To identify the independent variables of GS, linear regression was employed for univariate and multivariate analyses in CAD subjects. Variables of the statistical significance in univariate analysis and variables relevant with risk factors were enrolled in the multivariate linear regression analysis (Table 4). Table 4 showed that relationship of MPV and GS could be adjusted by other conceivable confounders, including gender, smoking status, diabetes, HbA1c, fibrinogen, WBC, TC, LDL-C, non-HDL-C, and apoA1. As a result, MPV was still independently positively correlated with GS after the adjustment (β = 0.171, *p* < 0.001, Table 4). Furthermore, we found an apparent trend of elevating GS by MPV tertiles (Figure 3).

**Table 4:** Association of MPV with GS (GS>0, *n* = 4,425).

**Variables**	**Univariate**		**Multivariate**	
	**β**	***P*-value**	**β**	***P*-value**
Male	0.105	<0.001	0.059	0.005
Age	−0.005	0.723	0.025	0.135
Smoking	0.071	<0.001	0.027	0.177
Hypertension	−0.001	0.940	0.016	0.295
Diabetes mellitus	0.098	<0.001	0.042	0.039
HbA1c	0.131	<0.001	0.066	0.001
hsCRP	0.146	<0.001	0.006	0.751
FIB	0.204	<0.001	0.093	<0.001
WBC	0.201	<0.001	0.083	<0.001
TC	0.145	<0.001	1.300	0.006
TG	0.021	0.169	0.036	0.200
LDL-C	0.190	<0.001	0.129	0.006
HDL-C	−0.136	<0.001	−0.236	0.102
non-HDL-C	0.184	<0.001	1.317	0.004
apoA1	−0.187	<0.001	−0.141	0.010
apoB	0.194	<0.001	−0.044	0.619
PDW	0.150	<0.001	−0.056	0.109
MPV	0.176	<0.001	0.171	<0.001

*Univariate linear regression analyses were first performed, from which variables with p < 0.05 or well-known risk factors were included for multivariate regression analyses. GS, Gensini score; hsCRP, high sensitivity C-reactive protein; FIB, fibrinogen; WBC, white blood cell count; TC, total cholesterol; TG, triglyceride; LDL-C, low density lipoprotein-cholesterol; HDL-C, high-density lipoprotein-cholesterol; non-HDL-C, non-high-density lipoprotein-cholesterol; apo, apolipoprotein; PDW, platelet distribution width; MPV, mean platelet volume*.

### Interaction Between MPV and Lipids

The CAD population was grouped based on MPV tertiles (tertile 1, MPV ≤ 10.4 fL; tertile 2, MPV 10.5–11.4 fL; tertile 3, MPV ≥ 11.5 fL) to investigate the relationship between MPV and lipid parameters (Table 5). Atherogenic lipids except TG were all positively and significantly relevant with MPV levels despite the use of statin (*p* < 0.001). On the contrary, HDL-C and apoA1 significantly decreased with MPV tertiles regardless of statin treatment. Synthetically considering the meaningful lipid parameters, the calculated lipid index increased with MPV tertiles in both the statin status groups (*p* < 0.001).

**Table 5:** Statin-stratified lipid levels by MPV in CAD, adjusted by age and gender.

	**MPV tertiles, (fL)**			***P*-value**
	**≤10.4** **(*n* = 1,486)**	**10.5–11.4** **(*n* = 1,549)**	**≥11.5** **(*n* = 1,390)**	
Not on statin (*n* = 1,197)				
TC, mmol/L	4.32 ± 0.98^a^	4.62 ± 1.01^b^	4.77 ± 1.20^b^	<0.001
TG, mmol/L	1.90 ± 1.04	1.98 ± 1.25	1.98 ± 1.31	0.547
LDL-C, mmol/L	2.83 ± 0.94^a^	3.12 ± 0.92^b^	3.27 ± 1.03^b^	<0.001
HDL-C, mmol/L	1.12 ± 0.32	1.10 ± 0.30	1.07 ± 0.28	0.079
non-HDL-C, mmol/L	3.20 ± 0.98^a^	3.52 ± 0.99^b^	3.69 ± 1.19^b^	<0.001
apoA1, g/L	1.30 ± 0.27	1.28 ± 0.26	1.25 ± 0.24	0.022
apoB, g/L	0.87 ± 0.24^a^	0.93 ± 0.24^b^	0.97 ± 0.28^b^	<0.001
Lipid index	1.17 [-0.73-2.94]^A^	1.99 [0.15-3.43]^A^	1.97 [0.37-3.73]^B^	<0.001
On statin (*n* = 3,228)				
TC, mmol/L	3.48 ± 0.85^a^	3.55 ± 0.86^b^	3.74 ± 1.05^c^	<0.001
TG, mmol/L	1.82 ± 1.45	1.91 ± 1.50	1.91 ± 1.45	0.305
LDL-C, mmol/L	1.99 ± 0.71^a^	2.05 ± 0.72^b^	2.24 ± 0.88^b^	<0.001
HDL-C, mmol/L	1.08 ± 0.31	1.07 ± 0.30	1.06 ± 0.32	0.375
non-HDL-C, mmol/L	2.40 ± 0.82^a^	2.48 ± 0.86^b^	2.68 ± 1.04^c^	<0.001
apoA1, g/L	1.26 ± 0.26^a^	1.25 ± 0.24^a^	1.23 ± 0.25^b^	0.003
apoB, g/L	0.68 ± 0.19 ^a^	0.69 ± 0.20 ^a^	0.70 ± 0.21 ^b^	<0.001
Lipid index	−1.29 [-2.50-0.21]^A^	−1.01 [-2.42-0.36]^B^	−0.82 [-2.31-0.98]^C^	<0.001

*One-way ANOVA or Kruskal–Wallis tests were performed to evaluate the potential differences within groups after the adjustment for age and gender according to the use of statin. Data were expressed as mean ± standard deviation or median [quartiles]. Differences in the same rows were shown by different lower-case letters (Bonferroni, P < 0.05) or capital letters (Games-Howell, P < 0.05). MPV, mean platelet volume; TC, total cholesterol; LDL-C, low density lipoprotein-cholesterol; HDL-C, high-density lipoprotein-cholesterol; non-HDL-C, non-high-density lipoprotein-cholesterol; apo, apolipoprotein*.

### Relation of Lipid Index With GS According to MPV

**Figure 4:**
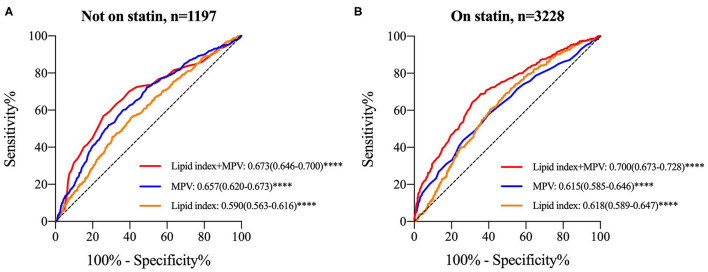
ROC curves for lipid index, MPV and “lipid index+MPV” combination to determine high GS stratified by statin use. **(A)** ROC curve in subjects without statin therapy; **(B)** ROC curve in subjects with statin treatment. Data were expressed as AUC (95% CI) and ****P < 0.0001. ROC, receiver operating characteristic; GS, Gensini score; MPV, mean platelet volume; AUC, area under ROC curve; 95% CI, 95% confidence intervals.

Lipid index was grouped into tertiles [low (≤-1.39), median (−1.38 to 0.90), and high (≥0.91)], and it was interacted with MPV tertiles in each GS tertile. Table 6 shows the multivariant logistic regression with adjustment for age, gender, smoking, diabetes mellitus, HbA1c, fibrinogen, and WBC. For patients without statin medications, the adjusted OR with 95% CI of low lipid index patients for low GS in the highest tertile of MPV was 2.436 (1.045-5.680). Nevertheless, no other modified risks were statistically significant. In the cohort on statin treatment, the data suggested a trend toward significance in patients with median and high GS. The adjusted OR (95% CI) for median GS in the upper tertile of MPV rose from 1.463 (1.063–2.014) in median lipid index group to 1.918 (1.301–2.828) in the high index group, whereas the OR (95% CI) for high GS was 2.068 (1.552–2.756) and 1.764 (1.219–2.551), respectively in the latter two lipid index groups. In addition, Figure 4 displayed the ROC curves of lipid index, MPV, and two features combination to predict high GS according to statin use. For individuals without statin medications, lipid index+MPV combination had a better predictive performance than the separate variable [AUC (95%CI) for lipid index, MPV, and lipid index+MPV was 0.590 (0.563–0.616), 0.657(0.620–0.673), and 0.673(0.646–0.700), respectively]. Similar results were also seen in individuals with statin [AUC (95%CI) for lipid index, MPV, and lipid index+MPV was 0.618(0.589–0.647), 0.615(0.585–0.646), and 0.700(0.673–0.728), respectively].

**Table 6:** Relation between lipid index and GS tertiles by MPV levels.

	**MPV tertiles, fL**			***P*-value**
	**≤10.4** **(*n* = 1,486)**	**10.5–11.4** **(*n* = 1,549)**	**≥11.5** **(*n* = 1,390)**	
**Not on statin (*****n*** **=** **1,197)**				
**Low GS (≤19)**				
Low lipid index	Reference	1.097 (0.820–1.467)	1.223 (0.877–1.707)	0.426
Median lipid index	Reference	0.807 (0.376–1.731)	1.249 (0.496–3.148)	0.582
High lipid index	Reference	1.343 (0.670–2.691)	2.436 (1.045–5.680)	0.039
**Median GS (20–48)**				
Low lipid index	Reference	0.912 (0.531–1.567)	1.242 (0.896–1.723)	0.537
Median lipid index	Reference	1.054 (0.484–2.297)	1.264 (0.541–2.956)	0.589
High lipid index	Reference	0.859 (0.420–1.756)	1.293 (0.595–2.810)	0.517
**High GS (≥49)**				
Low lipid index	Reference	0.885 (0.390–2.012)	1.375 (0.612–3.088)	0.584
Median lipid index	Reference	1.632 (0.937–2.843)	1.684 (0.961–2.952)	0.112
High lipid index	Reference	1.084 (0.754–1.561)	1.366 (0.955–1.953)	0.203
**On statin (*****n*** **=** **3,228)**				
**Low GS (≤19)**				
Low lipid index	Reference	0.734 (0.439–1.094)	0.775 (0.493–1.281)	0.396
Median lipid index	Reference	0.918 (0.668–1.261)	0.989 (0.725–1.349)	0.597
High lipid index	Reference	1.098 (0.749–1.609)	1.006 (0.679–1.490)	0.633
**Median GS (20–48)**				
Low lipid index	Reference	1.044 (0.746–1.459)	1.136 (0.782–1.722)	0.468
Median lipid index	Reference	1.058 (0.769–1.457)	1.463 (1.063–2.014)	0.020
High lipid index	Reference	1.729 (1.145–2.609)	1.918 (1.301–2.828)	<0.001
**High GS (≥49)**				
Low lipid index	Reference	1.172 (0.906–1.516)	1.168 (0.893–1.528)	0.395
Median lipid index	Reference	1.506 (1.135–1.999)	2.068 (1.552–2.756)	<0.001
High lipid index	Reference	1.539 (1.047–2.263)	1.764 (1.219–2.551)	0.009

*Multivariate logistic regression was performed to assess the interaction of lipid index and MPV in patients with GS tertiles, adjusted by age, gender, smoking, diabetes mellitus, HbA1c, fibrinogen and WBC. GS, Gensini score; MPV, mean platelet volume; WBC, white blood cell count. Data represent odds ratios with 95% confidence intervals*.

### Mediation Effects of MPV in the Relationship Between Lipids and GS

In the above section, we detected a tendency that the cohort with high GS had both higher lipid index and MPV than those with lower GS. For further probing of the correlation, we performed mediation analysis with MPV as mediators for the relationship of atherogenic lipids and GS. In this model, the independent variables were TC, LDL-C, non-HDL-C, apoB, and lipid index; GS was the dependent variable; and age and gender were included as covariates (Table 7). For patients not on statin, data indicated that MPV accounted for 20.07% mediation effect size in the relationship of lipid index and GS, whereas the effect size rose to 20.71% in patients with statin medications. Besides, MPV also had meaningful mediation effect sizes in lipids parameters and GS among patients without statin (proportion of MPV effect was 19.63% in TC, 16.99% in LDL-C, 18.80% in non-HDL-C, and 15.46% in apoB), whereas in patients on statin treatment, the effect size of MPV were 16.75% in TC, 13.17% in LDL-C, 12.62% in non-HDL-C, and 0.33% in apoB.

**Table 7:** Mediation analysis for lipid parameters and GS *via* MPV.

**Dependent variables**	**Total effect** **(*P*-value)**	**Direct effect** **(*P*-value)**	**Indirect effect** **(Bootstrap 95% CI)**	**Effect size (%)**
Not on statin (*n* = 1,197)				
TC	6.58 (*P* < 0.001)	5.29 (*P* < 0.001)	1.29 (0.767–1.931)	19.63
LDL-C	8.58 (*P* < 0.001)	7.12 (*P* < 0.001)	1.46 (0.856–2.200)	16.99
non-HDL-C	7.07 (*P* < 0.001)	5.74 (*P* < 0.001)	1.33 (0.785–1.966)	18.80
apoB	31.11 (*P* < 0.001)	26.30 (*P* < 0.001)	4.81 (2.650–7.086)	15.46
Lipid index	2.42 (*P* < 0.001)	1.94 (*P* < 0.001)	0.49 (0.280–0.737)	20.07
On statin (*n* = 3,228)				
TC	5.67 (*P* < 0.001)	4.72 (*P* < 0.001)	0.95 (0.641–1.294)	16.75
LDL-C	8.74 (*P* < 0.001)	7.59 (*P* < 0.001)	1.15 (0.802–1.577)	13.17
non-HDL-C	7.53 (*P* < 0.001)	6.58 (*P* < 0.001)	0.95 (0.648–1.302)	12.62
apoB	33.66 (*P* < 0.001)	29.59 (*P* < 0.001)	4.00 (2.678–5.699)	0.33
Lipid index	1.69 (*P* < 0.001)	1.34 (*P* < 0.001)	0.35 (0.231–0.480)	20.71

*The mediation analyses were performed with MPV as the mediator, lipids as independent variables and GS as the dependent variable. GS, Gensini score; MPV, mean platelet volume; 95% CI, 95% confidence intervals; TC, total cholesterol; LDL-C, low density lipoprotein-cholesterol; non-HDL-C, non-high-density lipoprotein-cholesterol; apo, apolipoprotein*.

## Discussion

Previous studies have proved that dyslipidemia contributes to the development of atherosclerosis, but the significance of high MPV in CAD remains controversial. As far as we know, this is the research that evaluated the interaction between lipids and MPV in the severity of CAD for the first time. In summary, the major innovation of our studies is the discovery of the relationship between atherogenic lipids and GS partly mediated by MPV, of which the effect size reaches 20.71 and 21.07% in individuals on or not on statins, respectively.

In this study, we measured the correlation in lipid parameters, MPV, and GS pairwise. Our results verified that atherogenic lipids were risk factors of CAD, with a significant positive correlation between lipid index and GS. High MPV level was positively related to high GS, whereas the positive relationship between atherogenic lipids and MPV was also conformed. Furthermore, we found that the interactions of MPV and lipids was obvious in patients with high GS, especial those on statin. Such a relationship satisfied the premise of mediation analysis and it proved that MPV played a significant role in mediating lipids and GS.

It is widely acknowledged that TG, LDL-C, non-HDL-C, apoB, TC/HDL-C, and apoB/apoA1 were elevated in patients with high GS, which our research and previous studies have conformed. A variety of reliable evidence proved that LDL had a clear causal relationship in atherosclerosis (23). The concept of non-HDL-C was first proposed in 2001, and it might be better in CAD risk assessment than LDL-C (24). This result might be explained by the theory that non-HDL-C represented the sum of all atherogenic lipoproteins, or non-HDL-C could better express the exact level of LDL particles than LDL-C (25). In the forest plots, the predictive ability of apoB had a higher OR than other single lipid parameters. Over 90% apoB exists in LDL, representing the level of LDL (23). Some studies believed that apoB contained more information about the risk of CAD. The 2019 ESC/EAS guidelines for the management of dyslipidemias included for the first time apoB in the risk assessment of CAD and efficacy of lipid-lowering therapy (26). Multiple studies have confirmed that apoB is proportional to the occurrence of CAD (27, 28).

The HDL particles have certain effects of antioxidation, antiinflammatory, antithrombosis, which are contributors to protect plaque (29). Our study found that the HDL-C and apoA1 were negatively correlated with the severity of CAD, which could be due to the ability of HDL to transport cholesterol reversely, and with apoA1 remaining the major protein component of HDL (30, 31). A meta-analysis of the four large prospective studies, including the Framingham study, showed that for every 1 mg/dL increase in HDL-C, the risks of CAD in men and women reduced by 2 and 3%, respectively (32).

Li et al. found that a combination of lipid-related biomarkers had an advantage in predicting coronary severity compared with single biomarkers (33). Consistent with Li's study, the lipid index extracted by principal component analysis could better express serum lipids in our cohort.

Platelets play the role of key regulators of the endothelia damage and the rupture of vulnerable plaque in atherosclerosis, contributing to an excessive platelet activation (11). MPV is an important and simple marker, which significantly increase during platelet activation by expanding pseudopodia and a high platelet turnover stimulating the bone marrow to produce more platelets (18, 34, 35). Murat et al. first found a positive association between high levels of MPV and severity of CAD by Gensini and Syntax score in 2013 (36). This finding was also reported by Ekici et al. (37) and Vogiatzis et al. (38). Further research showed that MPV could reflect the stenosis of coronary arteries that were influenced by chronic inflammation and diabetes (39). These results are in accordance with our study. However, Tavil et al. did not find a clear relationship between MPV and the severity of CAD (40), and a large prospective study (*n* =1,411) also drew an opposite conclusion (41). It may be attributed to the acute fluctuation of MPV in patients with CAD (42). The impact of widely used antiplatelet medications cannot be neglected, especially in patients who have just been admitted to the hospital with loading dose due to acute coronary syndrome, which has a certain effect on the measurement of platelet parameters.

There may be multiple mechanisms involved in the association between high MPV and the severity of CAD. Thus, we employed a multivariate linear regression analysis to rule out the potential cofounders. In addition to the lipid indicators that we mainly studied in this model, we found that fibrinogen and WBC were independent risk factors. Fibrinogen and WBC are both inflammatory biomarkers and play crucial roles in platelet aggregation (43), which was reported as high sensitivity in CAD prognosis and monitoring in many studies (44, 45). Platelets can remain activated in coronary plaque for a longer period than that in the peripheral circulation accompanied by secreting a variety of chemokines and cytokines, which constitutes a positive feedback loop of inflammatory progress (46).

There are a few studies on the relationship between traditional lipid parameters and MPV in CAD. Varol et al. found that MPV was significantly elevated in patients with HDL-C < 1.94 mmol/L (35 mg/dL) (47). Jeon et al. found that patients with increased lipoprotein(a) tend to have high levels of MPV (48). Statin treatment is known to have lipid-lowering function. Besides, some studies also showed statin-modulated platelet activation, including MPV. A pilot study in the cohort of 40 hypercholesterolemia patients treated with atorvastatin 40 mg/day exhibited reduction of proatherogenic lipid profiles, especially small dense (sd)-LDL-C as well as MPV (49). Similar results were also reported by Akyüz et al. (50). A meta-analysis demonstrated that statins including atorvastatin and rosuvastatin could significantly reduce MPV, irrespective of cholesterol levels (51). The possible non-lipid-related mechanisms of direct platelet membrane interactions and several signaling pathways such as NO synthase activity may contribute to decreasing MPV (52–54). In our study, the levels of atherogenic lipids and MPV were lower in on-statin group, whereas the interactions represented by OR and mediation effects of lipids and MPV were higher than not-on-statin group. This may be interpreted to indicate that although statin reduced proatherogenic lipids and MPV, the risk of coronary stenosis progressively increased due to severe platelet reactivity for those who had high MPV levels after statin treatment (55). The results suggested that we may lay concerns on the persisted residual risk of MPV after lipid lowering therapies, especially in individuals at high risk of cardiovascular events. Numerous *in vitro* assays demonstrated that lipids activated platelets by inflammatory factors, whereas platelet activation was triggered by oxidative stress, receptors of peptide hormones, signaling proteins, microRNAs, etc., which further affected the activation process (56). The *in vitro* study by Carnevle et al. showed that platelets could oxidize LDL into oxidized LDL (ox-LDL) through NADPH oxidase 2 (57), whereas ox-LDL enhanced activation of platelets through receptors such as CD36 and LOX-1 (58).

## Limitations

There are several limitations within our study. First, the results shown should be interpreted with caution due to the single-center and cross-sectional nature of the study. Imbalanced gender ratio in the cohort may have potential impact, but it was consistent with Chinese epidemiological results of male as risks for CAD (59). The variables we selected were incomplete and there might be more cofounders. Besides, follow-up data after discharge was not available to determine the relationship between MPV and prognosis. More researches are required to assess whether our data can be extended to larger populations. Second, we only selected MPV as the indicator of platelet activation in traditional platelet parameters. However, the measurement of platelet parameters was influenced by the vacutainer and its anticoagulant. We used EDTA as a chelator, which swells platelets time dependently (42). Our hospital requires a fast determination of blood sample within 1.5 h to avoid bias as much as possible. Third, the cohort of non-CAD group contained subjects suspected of having CAD, and they might have other diseases such as organic heart disease, lung disease, etc. Last but not least, we did not reveal the molecular biological mechanisms and pathogenesis in the current study.

## Conclusion

In summary, we firstly employed mediation analysis to examine the role of MPV in the relationship of lipids and GS. The mediating effect size of MPV reached around 20%. We may give greater attention to the residual risks of MPV after statin treatments in high-risk CAD individuals. Our findings may have some implications for clinical practice of high MPV and for future studies of the biological process by which lipids activate platelets.

## Data Availability Statement

The raw data supporting the conclusions of this article will be made available by the authors, without undue reservation.

## Ethics Statement

The studies involving human participants were reviewed and approved by Medical Ethics Committee of Zhongshan Hospital. The patients/participants provided their written informed consent to participate in this study.

## Author Contributions

QL and YY designed the study. YY, XL, and QX enrolled the subjects and collected the data. YY, ZW, and QJ analyzed the data. YY prepared the manuscript. XL revised the manuscript and gave modification advises. All authors contributed to the article and approved the submitted version.

## Conflict of Interest

The authors declare that the research was conducted in the absence of any commercial or financial relationships that could be construed as a potential conflict of interest.

## Publisher's Note

All claims expressed in this article are solely those of the authors and do not necessarily represent those of their affiliated organizations, or those of the publisher, the editors and the reviewers. Any product that may be evaluated in this article, or claim that may be made by its manufacturer, is not guaranteed or endorsed by the publisher.
