# Supplementary material for: Interaction of Lipids, Mean Platelet Volume, and the Severity of Coronary Artery Disease Among Chinese Adults: A Mediation Analysis
Source: Front Cardiovasc Med. 2022 Jan 31;9:753171. doi: 10.3389/fcvm.2022.753171 (PMC8841779; doi:10.3389/fcvm.2022.753171)
Supplement: Supplementary file 1 [file Table_1.DOCX]

Baseline demographic and clinical characteristics of CAD subjects categorized by GS tertiles

|  | GS tertiles | | | P value |
| --- | --- | --- | --- | --- |
|  | low (≤19)  (n=1495) | median (20-48)  (n=1490) | high (≥49)  (n=1440) |  |
| **Risk factors** | |  |  |  |
| Age (years) | 65.01±9.58 | 64.81±10.40 | 65.01±11.19 | 0.835 |
| Male, n (%) | 1031 (69.0%) | 1151 (77.2%) | 1173 (81.5%) | <0.001 |
| Smoking, n (%) | 708 (47.4%) | 866 (58.1%) | 845 (58.7%) | <0.001 |
| BMI (kg/m^2^) | 24.70±3.57 | 24.68±3.54 | 24.76±3.63 | 0.803 |
| Hypertension, n (%) | 685 (45.8%) | 683 (45.8%) | 658 (45.7%) | 0.996 |
| Diabetes mellitus, n (%) | 430 (28.1%) | 565 (37.9%) | 580 (40.3%) | <0.001 |
| **Laboratory values** | |  |  |  |
| HbA1c, mmol/mol | 6.21±1.08 | 6.50±1.29 | 6.64±1.39 | <0.001 |
| hsCRP, mg/L | 0.96 (0.44-2.71) | 1.51 (0.58-4.83) | 2.86 (0.95-9.90) | <0.001 |
| FIB (g/L) | 270.40 (236.00-318.68) | 291.20 (248.08-347.45) | 311.70 (263.30-379.53) | <0.001 |
| WBC, 10^9^/L | 6.21 (5.19-7.50) | 6.85 (5.63-8.35) | 7.45 (5.95-9.33) | <0.001 |
| PLT, 10^9^/L | 202.40±57.71 | 210.87±61.38 | 208.04±60.54 | <0.001 |
| PCT, % | 0.22±0.056 | 0.22±0.057 | 0.22±0.058 | 0.002 |
| MPV, fL | 10.87±1.09 | 10.89±1.03 | 11.20±1.07 | <0.001 |
| PDW, % | 13.05±2.51 | 12.80±2.31 | 12.94±2.38 | 0.055 |
| cTnT, ng/mL | 0.009 (0.006-0.015) | 0.012 (0.073-0.034) | 0.026 (0.011-0.31) | <0.001 |
| TC, mmol/L | 3.69±0.95 | 3.78±1.01 | 4.09±1.18 | <0.001 |
| TG, mmol/L | 1.86±1.34 | 1.89±1.34 | 1.96±1.53 | 0.147 |
| LDL-C, mmol/L | 2.15±0.82 | 2.30±0.89 | 2.62±1.05 | <0.001 |
| HDL-C, mmol/L | 1.13±0.33 | 1.07±0.29 | 1.02±0.28 | <0.001 |
| non-HDL-C, mmol/L | 2.56±0.92 | 2.71±1.00 | 3.06±1.15 | <0.001 |
| apoA1, g/L | 1.32±0.26 | 1.24±0.24 | 1.20±0.24 | <0.001 |
| apoB, g/L | 0.71±0.21 | 0.75±0.23 | 0.83±0.27 | <0.001 |
| **Cardiovascular medication use** | |  |  |  |
| Beta-blocker, n (%) | 761 (50.9%) | 774 (51.9%) | 747 (51.9) | 0.817 |
| ACEI/ARB, n (%) | 296 (19.8%) | 355 (23.8%) | 363 (25.2%) | 0.001 |
| Statin, n (%) | 1150 (76.9%) | 1112 (74.6%) | 966 (67.1%) | <0.001 |

Data were expressed as n (%), median (quartiles) or mean±SD. P values <0.05 were statistically significant. CAD, coronary artery disease; GS, Gensini score; BMI, body mass index; hsCRP, high sensitivity C-reactive protein; FIB, fibrinogen; WBC, white blood cell count; PLT, platelet count; PCT, plateletcrit; MPV, mean platelet volume; PDW, platelet distribution width; cTnT, cardiac troponin T; TC, total cholesterol; TG, triglyceride; LDL-C, low density lipoprotein-cholesterol; HDL-C, high-density lipoprotein-cholesterol; non-HDL-C, non-high-density lipoprotein-cholesterol; apo, apolipoprotein; ACEI, angiotensin converting enzyme inhibitors; ARB, angiotensin receptor blocker.
